# Osseous Structure of the Teeth

**Published:** 1882-06

**Authors:** A. F. McClain


### OSSEOUS STRUCTURE OF THE TEETH.

### Pathology of the Osseous Structure of the Teeth in Pregnancy.

BY A. F. McCLAIN, M.D., D.D.S.

In view of the illimitable scope, together with the indefinite character of the section which has been assigned the committee, which I have the honor to represent on this occasion for treatment, I thought perhaps that, by directing our inquiries to a special matter connected with pathology, it would prove more interesting, if not more profitable, than to discuss pathology or surgery in a general sense, as it otherwise would have been necessary to do. Hence, I have chosen for a theme, "Pathology of the Osseous Structure of the Teeth as influenced by Pregnancy."

It must be within the observation of every practitioner of the dental art, that, notwithstanding the lights our boasted attainments have furnished, every effort made toward the preservation of the dental organs in that condition has resulted in utter and humiliating failure.

The local symptoms usually attending the period of gestation may vary in different cases so far as regards the quantity of the salivary and mucous secretions; but a strong acid reaction is found to be an invariable accompaniment of the disorder which is manifested in acute sensibility about the necks of the teeth, followed by erosions near the margin of the gums, softening of the enamel, and ultimately by the rapid disintegration of the dentine.

Now, although the pathological phenomena are frequently manifested in other conditions than that referred to, being often met with in young persons of either sex, and especially in individuals of dyspeptic habits, or in those addicted to the use of mineral or strong vegetable acids, still the almost universality of their occurrence during gestation, and the disappearance of the general symptoms when that period has once been passed, indicates it would seem, pretty clearly that the disorder owes much of its virulence to symtomatic causes.

The extent of local lesions in any case is governed very much by the structural quality of the teeth, and by their hereditary predispositions; hence it may naturally be deduced that the least appreciable abnormal dentinal changes occur in those cases where dense, perfect dental organisms exist, in connection with perfect general systemic health, whereby those depressing influences which tend to weaken the resisting capacity of the organisms are obviated.

The sympathetic influences which a gravid uterus exerts upon the stomach in producing disordered gastric secretions being an established fact, it is readily seen how the oral fluids, on becoming vitiated through chemical reactions and nervous relations of organs, conjoined to the operations of predisposing causes, the destructive processes become greatly accelerated by the augmented solvent properties of such products.

Of predisposing causes, debilitating influences, hereditary constitution, temperament, etc., have been classed by physiologists as being the most efficient. Now, how far temperament conduces to abnormal functions, aside from the control it has naturally in influencing the quality of the texture, is not positive. But we do know that in certain temperaments the nervous, for instance, irritability of the excito-motor system exists to a high degree, and that this natural nervous irritability, as modified by certain temperaments, and manifested through the sympathetic system, is susceptible under the play of mental emotions of so altering or perverting the normal constituents of the secretions as to render them positively poisonous. The pernicious effects of nourishing infants from the breast when the mothers are laboring under great mental excitement are too well established to render it necessary to cite authorities in proof of that fact.

Again: in erosion of the enamel, the microscope having failed to reveal capillaries whereby the sanguineous fluids might be transmitted, or the presence of nerves, the process, therefore, cannot be attributed to interstitial absorption, as neither nutritive nor destructive assimilation obtains in the enamel.

The cause of the rapid dissolution of the teeth during gestation has been generally ascribed to defective nutrition; in that, the food lacking in the elements of ossific matters, the osseous system of the new organism in process of building makes demands which can be supplied only by the osseous system of the mother.

On the other hand, it is urged against this hypothesis, that two women, placed under similar circumstances, and having similar temperaments, and fed at the same table, partaking of the same food, will present conditions diametrically opposite, the one being in robust health, comparatively free from the usual sympathetic disturbances, whilst the other will exhibit all the symptoms described. in their most aggravated forms. The fact is, human knowledge on this subject is limited, or, in other words, is hypothetical. That the offspring *in utero* actually preys upon the bony textures of the parent for those earthy constituents necessary for its own structures is not sufficiently proven to justify the acceptance of the theory as a philosophical truth. Neither is it to be assumed that the seemingly contradictory symptoms presented in the instances cited, an absorptive process existed in one case and not in the other, or that the difference was due to a difference in sympathetic relations of the organs.

Now, the rational deduction to be drawn from these facts is, that the assimilative functions and vital forces in one case were superior to those of the other; whilst the latter, in being unable to appropriate sufficiently of the earthy principles from her food to supply her own wants and those of the new being, her vital forces were impared, whilst at the same time the sympathetic influences operating from the pregnant state, so vitiated or altered the gastric and oral secretions as to induce chemical reactions, which, in connection with other exciting causes, produced the lesions presented.

Having shown, or attempted to show, that the pathological phenomena in this condition were due to physico-vital causes, the obvious treatment in such cases consists in employing all those hygienic means best calculated to build up the system, and control waste, such as generous, wholesome diet, fresh air, exercise, mild tonics, and the exhibition of nervous sedatives when their use is indicated. Above all, are the assimilative functions to be encouraged by the administration of nutritive stimulants, of which nothing has given more satisfactory results in cases where they have been tried, than the soluble phosphate of lime preparations; for instance, the hypophosphites, particularly the *lacto-phosphate*, given in doses of from one to three grains, according to circumstances, at each meal. Not unfrequently the most urgent dentinal tenderness and odontalgic symptoms, as well as the tendency to decalcification of the teeth, will entirely subside after the exhibition of this drug has been followed for a few days.

This mode of medication commends itself as much for the beneficial effects derived by the mother as for the tendency it possesses of imparting to the child good dental structures. And especially is the same course indicated toward the child, if an hereditary predisposition to bad teeth exists in the mother, exhibiting it with the view, not only of improving the structural quality of its teeth, but also to mitigate the ills which are common to the period of *teething*—a virtue this remedy possesses to an eminent degree, as I have had occasion to witness. Its administration should begin early after birth, by dissolving the lacto-phosphate in the milk given from the bottle, and to continue its use in the ordinary food until after the first dentition shall have been completed. Minimum doses are preferable, inasmuch as when larger quantities are given, a waste of the medicine is incurred, in consequence of the system not appropriating more than a certain amount. One-grain doses are therefore sufficient for all practical purposes.

Of course, in women, the local treatment should not, in the meantime, be neglected. To this end, the mouth should be rinsed with diluted lime-water or a solution of bi-carbonate of soda three or four times a day, and at night, just before retiring to rest, to pack precipitated chalk about the necks of the teeth, for the purpose of neutralizing the acids that are so apt to accumulate at the margin of the gums. It is almost superfluous to mention, in this connection, the necessity which exists for the exercise on the part of the dental surgeon of some judgment in the performance of his duties, by avoiding long and exhausting operations in these cases, especially in advanced stages of pregnancy, or when there is debility, or where miscarriages have heretofore occurred.

Here plastic fillings, such as gutta-percha or the pyro-phosphate of zinc stoppings, become excellent temporary expedients for arresting the ravages of caries, until the patient's condition be such as to admit of permanent operations being performed.

DISCUSSIONS.

THE PRESIDENT. We have the theoretical knowledge, but we have not had the practical, so as to be able to give personal experience in the matter.

DR. McLAIN. When I first commenced the use of lacto-phosphate of lime, some ten or twelve years ago, it was in this way. There was a physician whose family was afflicted with bad teeth. He had neglected his, and his wife and all of his first children had bad teeth; and I suggested something of this kind in a soluble form. He began to use the preparation, and to give it to the mother, and the children that were born afterwards had no dental trouble at all; he continued the remedy even after the child had received his second dentition. Before I left New Orleans he brought the boy, then ten or twelve years old, for me to see his teeth, which were perfect, and did not have a blemish. And the boy's limbs were like a giant's. Now, our medical authorities all contend that real phosphates of lime are not appropriated by the system, but are taken into the stomach, and pass out unchanged. This matter has been tested by two eminent professors in Paris, who have been experimenting with lacto-phosphate of lime a long time. They tried it on Guinea-pigs, feeding them all alike, but giving some of them lacto-phosphate of lime, and some of them none. When they were killed, it was found that the bones of those which had had the lacto-phosphate of lime would weigh 33⅓ per cent. more than the others, showing that it was appropriated by the system.

In the matter of pains, it is a sovereign remedy. I have had occasion to administer it in San Rafael to a woman who had been suffering pain two days, and the administration cured it. Now, about the theory of the new being, the embryo, preying on the bony tissues of the mother; I think it is not true, although she has to supply all the demands; consequently, if she does not get enough in her food to supply that demand, I think in such cases this should be administered.

Dr. A. Warner. In selecting the substance from which you prepare this, is it always done from calcined bone, or some from quick-lime?

Dr. McLain. They take calcined bone, combined with lactic acid, so as to form a thick paste.

Dr. Robinson. We discussed this matter some years ago, and our minutes made me say a great many ridiculous things I did not say, nor did anybody else. Consequently, we will have to be very careful, or we will get the thing mixed again. I have been investigating one point a great deal, which is in regard to whether the human system assimilates directly from the mineral kingdom; and I must say I don't think I know as much about it as when I started, although I have the views of thirty or forty men who think they know all about it, and rank high as scientists. There is a common opinion among doctors, dentists, and others, that we can and do appropriate directly from the mineral world.

In a private correspondence with the editor of the *Ohio State Journal*, he said that animals did appropriate directly from the mineral world. He had taken a number of hens, to some he had given lime, and withheld it from others; and the hens that had the lime had the thickest shells on their eggs. When I asked him where he got the lime from, he said from calcined oyster shells, so he could not answer the question at all, but confirmed rather the other side. I have got all the wisdom of the annual report of a medical school here, and they answer my questions in the same way. The second time I asked the professor of physiology about it, he said that he had observed everything on the subject, and when he came to look it over, he said there is one thing he was dead sure of, that we took from the mineral kingdom, that was oxygen. I don't know that we all yield that point, but I hold that we have all got a great deal to learn about this thing. That matter has been so mixed in my mind, that I am a little surprised to see others seem to have it settled in theirs. Somebody burned some limestone and found that some animals did absorb it; but then if you take nearly all the limestone in the world, and if you look at it, you will find it is a formation from the animal world, it is nearly all made up of shells from the various extinct mollusks. Consequently, that lime has been prepared already from the animal system. When I take a cup of water into my system, does it undergo any chemical change? It goes in water, and comes out water. But as to whether the elements of water in my blood are in any different condition than the elements of water in their normal condition, I don't know. And as to this lime matter, the question is, when it goes into the system does it remain inorganic lime, or is it vitalized? I do not know, nor do I know that anybody else does.

I am told there is a work published by Dr. Gangee, of Manchester. I have written for it several times, but could not get it. If I could get that, I would steal his ideas and give the Association the benefit of them. I say that this point is of great interest to the dentists—"an ounce of prevention is better than a pound of cure," and if any man can give us something to prevent teeth from decaying, it may not be so much in the dentist's pocket, but it will be a grander gift to humanity.

Dr. W. A. Knowles. I think there is one example of an inorganic substance which can be taken into the system; that is chloride of sodium.

Dr. Robinson, There is no question about that; but the substance is not vitalized; it is not broken up and separated. We need it, and must have it; but it does not undergo a change.

Dr. S. E. Knowles. My friend, Dr. Robinson, does not, I think, clearly discriminate. He spoke of oxygen as being a mineral. I suppose he meant to refer to it as being an inorganic substance. There seems to be a rule in regard to the assimilation of matter by animals, something like this: In case a substance is assimilated at all by being made a part of an organized body, that it is best assimilated by an animal when it has previously passed through a vegetable. There are some crystallized substances, it is true, that appear to undergo a change by being made an integral part of an animal body. In case any substance is changed, although we may not be able by any test to note the fact of the change, it is necessary for it to pass through vegetable life before it can be assimilated as a part of the animal kingdom. In regard to the experiment spoken of, there was another. I do not know whether by the same gentleman or not, which tended to show the power of animals under certain circumstances, to assimilate a great quantity of phosphate of lime. The animals were fed on a like diet, only some had phosphate of lime added. The limbs of one or two were fractured, and others remained unbroken. At the end of a certain time the animals were all killed, and it was found that in those to which phosphate of lime had been administered the fracture was in a greater state of advance than in the others.

I have tried the experiment of administering lacto-phosphate of lime in the case of pregnant women who manifested an insufficiency of that substance in the teeth, and had very little success. The fault does not seem to be in regard to the pabulum, but it seems to be a want of nutrition. There is something back of it, but what it is I do not know.

Dr. McLain. As to this intimation of Dr. Knowles in regard to the experiment he spoke of, I got my information from the same account. It was fifteen years ago that they commenced those experiments, with the view of trying lacto-phosphate of lime under those conditions. That is where I got my idea. I will say furthermore, that I do not believe that the animal system can appropriate directly any inorganic substance; it must pass through a vegetable. In certain districts—take Kentucky, for instance—the men are large and robust, and possess a bony structure, and all the animals have the same characteristic. I do not conceive that they take that from the earth; but then lime exists in great abundance in that State, especially in the blue-grass region. I think they take that from the vegetable and the animal, too. The animal absorbs it browsing, and then the man gets it.

Dr. Harding. I have found by referring to memoranda I

have kept, that where women have raised the largest families, the children have the best teeth. It is a custom in this country for women, when they are pregnant, to keep in-doors, a custom that results in sour stomach. Now, take it where women labor in England, and eat oat meal, you will find good teeth. It is so in all parts of the world. There are regions in the south of Ireland where they rarely see meat. and a dentist would starve to death there. And, again, in the north of England, where they eat unleavened bread. Now, where we find a woman is constipated in that condition, the only way for her is to take exercise; and I know for a fact, that women in that condition are generally constipated.—*Transaction of Cal. State Dental Association.*

